# Noninvasive Evaluation of Angiogenesis and Therapeutic Response after Hindlimb Ischemia with an Integrin-Targeted Tracer by PET

**DOI:** 10.31083/j.rcm2312408

**Published:** 2022-12-14

**Authors:** Zhongchan Sun, Weibin He, Shuang Xia, Guang Tong, Lin Zeng, Ling Xue, Junqing Yang, Ning Tan, Pengcheng He

**Affiliations:** ^1^Department of Cardiology, Guangdong Provincial People’s Hospital, Guangdong Academy of Medical Sciences, 510080 Guangzhou, Guangdong, China; ^2^Guangdong Cardiovascular Institute, Guangdong Provincial People’s Hospital, Guangdong Academy of Medical Sciences, 510080 Guangzhou, Guangdong, China; ^3^Department of Cardiac Surgery, Guangdong Provincial Key Laboratory of South China Structural Heart Disease, Provincial People’s Hospital, Guangdong Academy of Medical Sciences, 510080 Guangzhou, Guangdong, China; ^4^Department of Cardiology, Heyuan People’s Hospital, 517000 Heyuan, Guangdong, China

**Keywords:** angiogenesis, PET imaging, peripheral arterial disease, hindlimb ischemia, integrin, RGD peptide

## Abstract

**Background:**

Peripheral arterial disease (PAD) can severely compromise limb vitality and function. Angiogenesis plays an important role in healing of ischemic lesions. Radiolabeled RGD (Arg-Gly-Asp) peptides specifically targeting α_v_β_3_ integrin are promising tracers for imaging angiogenesis. In this study, we investigated the application of a one-step labeled RGD in evaluation of angiogenesis and therapy response in a mouse model of hindlimb ischemia (HI) by positron emission tomography (PET).

**Methods:**

HI was induced by ablation of the femoral artery in mice. PET imaging using ^18^F-AlF-NOTA-PRGD2 (^18^F-PRGD2) tracer was performed at day 0 (pre-surgery) and days 3, 7, 14, and 21 after surgery to evaluate hindlimb angiogenesis longitudinally and noninvasively. The control peptide RAD (Arg-Ala-Asp) labeled with a similar procedure and a block agent were used to confirm the specific binding of ^18^F-PRGD2 to α_v_β_3_ integrin. *Ex vivo* CD31 staining was performed to detect angiogenesis. In addition, the angiogenic therapy response was monitored with ^18^F-PRGD2 tracer and immunofluorescence staining to confirm the imaging data.

**Results:**

The successful establishment of HI model was confirmed by ultrasound imaging and laser doppler perfusion imaging (LDPI). Specific binding of ^18^F-PRGD2 to α_v_β_3_ integrin was validated by minimal tracer uptake of the control peptide RAD and significant decrease of tracer accumulation when a block agent was added. Local accumulation of ^18^F-RRGD2 in ischemic hindlimb was detected as early as 3 days and reached a peak at 7 days after surgery. The temporal change of focal tracer uptake was positively correlated with the pattern of vascular density. Moreover, vascular endothelial growth factor (VEGF) treatment increased the tracer uptake and enhanced angiogenesis, which is consistent with integrin β3 expression.

**Conclusions:**

PET imaging of a one-step labeled tracer ^18^F-PRGD2 targeted to α_v_β_3_ integrin allows longitudinal monitoring of ischemia-induced angiogenesis and noninvasive assessment of VEGF treatment response in a mouse model of hindlimb ischemia. The simple synthesis procedure and *in vivo* performance of this PET tracer enables the feasibility of future clinical translation in ischemic cardiovascular diseases.

## 1. Introduction

As a leading cause of impaired limb viability, peripheral arterial disease (PAD) is a progressive atherosclerotic process by which blood vessel narrowing or occlusion occurs in the extremities [1]. Owing to poor blood perfusion, local ischemia can induce serial pathophysiological responses in skeletal muscle tissue [2], including tissue necrosis, inflammation, angiogenesis, and tissue regeneration [3, 4].

Angiogenesis, defined as new capillaries formation from preexisting vasculature, is mainly triggered by tissue ischemia or hypoxia [5]. Generation of new microvasculature is critical in the condition of PAD, as angiogenesis can promote the restoration of post-ischemic blood perfusion to skeletal muscle [6]. Therapeutic angiogenesis with the goal of stimulating new blood vessel formation within ischemic tissues, has received extensive attention for PAD treatment [7]. Therefore, the development of molecular imaging approaches to noninvasively monitor the recovery process of peripheral ischemic lesions would have significant clinical benefits.

Numerous factors have been involved in the angiogenic process in the setting of PAD [8]. Vascular endothelial growth factor (VEGF) has been recognized as one of the most effective stimuli to the development of vascular network [9]. Apart from VEGF, integrins also play a crucial role in the regulation of angiogenesis [10]. These transmembrane receptors are able to modulate cell adhesion, migration, and proliferation. Particularly α_v_β_3_ integrin has aroused great interest among researchers owing to its significant role in the regulation of endothelial cell migration and interplay with the extracellular matrix (ECM) during angiogenesis [11]. The α_v_β_3_ integrin has great abundance on the surface of the endothelium with high angiogenic activity. Thus, α_v_β_3_ integrin has become the primary target of many specific probes for noninvasive evaluation of angiogenesis, including arginine-glycine-aspartic acid (RGD)-containing peptides.

Radiolabeled RGD peptides, targeted at α_v_β_3_ integrin, have been widely utilized for angiogenesis imaging in tumor. However, angiogenesis not only occurs in cancer development but also is a critical contributor to the improvement and recovery of ischemic lesions. After myocardial ischemia/reperfusion (MI/R) injury, α_v_β_3_ integrin expression in cardiac tissue has been noninvasively monitored by numerous isotopes including ^99^mTc [12], ^111^In [13], ^125^I [14, 15] by single photon emission computed tomography (SPECT), and ^18^F [16] and ^68^Ga [17] by positron emission tomography (PET). In addition, previous studies also have used iodine-125 (^125^I)- and ^99^mTc-labeled [16] RGD peptides to visualize and quantify the activated angiogenesis post limb ischemia by targeting α_v_β_3_ integrin. Due to better imaging quality, higher diagnostic accuracy and lower injection doses, PET imaging has been more widely used for clinical imaging than SPECT imaging. Among multiple positron-emitting radioisotopes for PET imaging, ^18^F is ideal for the development of RGD peptide-based angiogenesis-targeted PET radiotracers as a result of its favorable physical properties [18].

Most approaches labeling RGD peptides with ^18^F involve multiple-step synthetic procedure [19], which is time-consuming and may hamper the widespread use of these ^18^F-labeled RGD tracers. With the development of molecular imaging, the labeling procedure can be simplified by linking RGD peptides with a pre-attached functional group, which contains an active component available for fluoride displacement [18, 20]. In our previous studies [21], we have applied a one-step labeled tracer ^18^F-AlF-NOTA-PRGD2 (^18^F-PRGD2) of integrin-targeted in a rat model of experimental MI/R. Owing to easy synthesis and high imaging quality, PET imaging using ^18^F-PRGD2 tracer achieved successful evaluation of angiogenesis in infarcted cardiac tissue after MI/R [21].

In this study, we established a mouse hindlimb ischemia (HI) model and microPET was performed with the ^18^F-PRGD2 tracer to assess α_v_β_3_ integrin expression level during angiogenesis progression in ischemic hindlimb. In addition, the angiogenic response to VEGF treatment was also monitored with this ^18^F-PRGD2 tracer.

## 2. Materials and Methods

### 2.1 Animals

To eliminate the potential impact of gender bias, exclusively male mice were used in the present study. FVB male mice were acquired from the Experimental Animal Center of Zhongshan Medical University, weighing 25–30 g and aged 8–9 weeks. All processes involving surgical operation and imaging scans were performed under anesthesia with isoflurane in oxygen (1.0–2.0%) with a delivery flow rate of 1.0 L/min. After surgery, meloxicam (10 mg/kg SC) was injected near the wound before mice completely awoke from anesthesia. Mice were sacrificed by cervical dislocation. All animal procedures were performed in accordance with the Guidelines for the Care and Use of Laboratory Animals and were approved by the Animal Ethics Committee of Guangdong Academy of Medical Sciences [22].

### 2.2 Hindlimb Ischemic Murine Model

After hair removal on both hindlimbs by depilatory cream, an incision was made through the skin of the thigh to expose the superficial arteries, veins, and nerves. After careful separation of the arteries, veins, and nerves, the main femoral artery and all branches in the right hindlimb were ligated and excised with femoral nerves carefully preserved. The similar procedure was performed in the left hindlimb except for the ligation and excision of the femoral artery, which was a sham operation and served as a control.

### 2.3 Laser Doppler Perfusion Imaging (LDPI) of Hindlimbs

Laser Doppler perfusion imaging (LDPI) was used to evaluate blood perfusion in preoperative and postoperative hindlimbs, respectively. After being anesthetized, mice were placed on a heating pad to maintain a stable body temperature, and were imaged using an analyzer (PeriScanPIM3 Perimed AB, Jakobsberg, Sweden).

### 2.4 Power Doppler Imaging (PDI) and Color Doppler Imaging (CDI) of Hindlimbs

To detect the blood flow in hindlimbs, power Doppler and color Doppler scans were performed with a Vevo 2100 imaging system (VisualSonics, Inc., Toronto, ON, Canada) before surgery and one day after surgery. After the induction of anesthesia, the vessel velocity and spatial distribution within the right hindlimb were monitored using a linear transducer in three-dimensional mode in power Doppler scans. Color Doppler mode in three-dimension was also applied to achieve an overview of blood flow as well as the flow direction by red and blue color spectrums in the right hindlimbs of pre-surgery and post-surgery, respectively.

### 2.5 Small Animal PET Imaging

As previously described [23], an Inveon small-animal microPET scanner (Siemens Preclinical Solutions) was used to perform PET imaging. After the induction of anesthesia, a single dose of 100 μL phosphate buffered saline (PBS) containing approximately 3.75 MBq (100 μCi) of ^18^F-AlF-NOTA-PRGD2 (^18^F-PRGD2) was injected via the tail vein. One hour after the tracer injection, static PET imaging scans were performed for 10 minutes. The acquired images were reconstructed with an algorithm, known as three-dimensional ordered subsets expectation maximization (3D-OSEM). ASI Pro VMTM software (Siemens Medical Solutions, Germany) was also used for image analysis. The ^18^F-PRGD2 accumulation within the ischemic hindlimb tissue was quantified by drawing regions of interest (ROIs) surrounding an entire limb on the coronal images in a three-dimensional manner. The mean radioactivity contained in the ROI divided by the dose administered to the animal gave the %ID per g (%ID/g). Then the tracer uptake of the ischemic right hindlimb divided by the tracer uptake of the contralateral left hindlimb gave the ^18^F-PRGD2 uptake ratio (ischemic/control).

### 2.6 Immunohistochemistry (IHC) Assay

Mice from various groups or at different time points after surgery were sacrificed and the skeletomuscular tissues of right hindlimbs were collected, underwent fixation in 4% paraformaldehyde solution, dehydration through graded solutions of ethanol, and paraffin embedding. Serial sections (5 μm thick) were cut and mounted on glass slides (p-45118, Fisher, Pittsburgh, PA, USA). The slides were then dewaxed, processed by microwave antigen retrieval, and subsequently incubated with 10% normal goat serum for 1 h and then overnight at 4 ℃ with CD31 antibody (mouse monoclonal, 1:100; #131M-9, Sigma, Burlington, MA, USA). The secondary antibody was anti-mouse IgG (#21538-M, Sigma, Burlington, MA, USA), which was detected with streptavidin–peroxidase complex and 0.1% of ${\mathrm{3,3}}^{\prime }$-diaminobenzidine (#D8001, Sigma, Burlington, MA, USA) in PBS with 0.05% $H_{2}$$O_{2}$ for 5 min at room temperature. In addition, hematoxylin and eosin (H&E) staining was also performed for tissue morphology analysis.

For quantification, skeletal muscle tissue of each mouse was divided into several parts, and about 10–20 slides were made from each sample. These slides were divided into two parts, one part for CD31 staining and the other part for integrin β3 staining. Capillaries (CD31 positive) density was determined by the average counts of 10 random microscopic fields, which was expressed as the number of capillaries per ${\mathrm{mm}}^{2}$.

### 2.7 Immunofluorescence Assay

To achieve double antibody staining, slides were concurrently incubated with both rabbit anti-integrin β3 (diluted 1:200; #ab179473, Abcam, Cambridge, MA, USA) and mouse anti-α-actin (skeletal) primary antibodies solution (diluted 1:250; #ab28052, Abcam, Cambridge, MA, USA). The combinations were visualized using a mixture of Cy3-conjugated anti-rabbit (#AP182C, Sigma, Burlington, MA, USA) and fluorescein isothiocyanate (FITC)-conjugated anti-mouse (#AP124F, Sigma, Burlington, MA, USA) secondary antibodies.

### 2.8 Experimental Protocols

In order to validate the success of HI model, PDI and CDI of the right hindlimb were performed before surgery and after surgery (n = 3), respectively.

To assess the binding specificity of ^18^F-PRGD2 to integrin receptor in ischemic tissue, twenty mice were randomly divided into four groups, including sham, HI, block and RAD. Each group contained 5 mice. In the sham group, both hindlimbs of mice were subjected to sham operation. Apart from the sham group, mice underwent HI surgery in the right hindlimb as well as sham operation in the left hindlimb in the other three groups. In both sham (sham, n = 5) and HI group (HI, n = 5), approximately 100 μCi ^18^F-PRGD2 was injected one hour before PET imaging scans on day 7 after surgery. Cyclic RGD peptide dimer ${\mathrm{E[c(RGDyK)]}}_{2}$ served as a blocking agent (18 mg/kg) and was injected 10 min before ^18^F-PRGD2 administration on day 7 after HI (block, n = 5). The control peptide RAD was synthesized by a similar procedure as RGD producing ^18^F-AlF-NOTA-RAD, which was injected via tail vein one hour before the PET scan on day 7 after HI (RAD, n = 5).

For *in vivo* imaging of angiogenesis induced by HI, PET scan was performed in a range of time points including day 0 (pre-surgery) and days 3, 7, 14, and 21 post surgery (n = 6).

For the treatment study, after the surgery of HI, a single dose of 100 μL PBS containing 0.5 μg VEGF was injected into ischemic gastrocnemius muscles below the site of occlusion at three different sites at 3 days and daily thereafter for three consecutive days (HI + VEGF, n = 5). The same amount of PBS without VEGF was administered in the same way at the same time points post ischemia (HI, n = 5). In addition, a sham group was also included on day 7 after sham operation (sham, n = 5).

Eight mice were sacrificed for histological staining. Thus, a total of 52 mice were used in the present study.

### 2.9 Statistical Analysis

Results are presented as the mean ± standard deviation (SD). Statistical analysis was performed using one-way analysis of variance (ANOVA) followed by the Bonferroni multiple comparison test. The correlation of tracer uptake ratio and angiogenic activity was examined by the Pearson correlation test. *p *< 0.05 was considered as statistically significant.

## 3. Results

### 3.1 Establishment of the Murine Model of Hindlimb Ischemia (HI) 

**Fig. 1.**
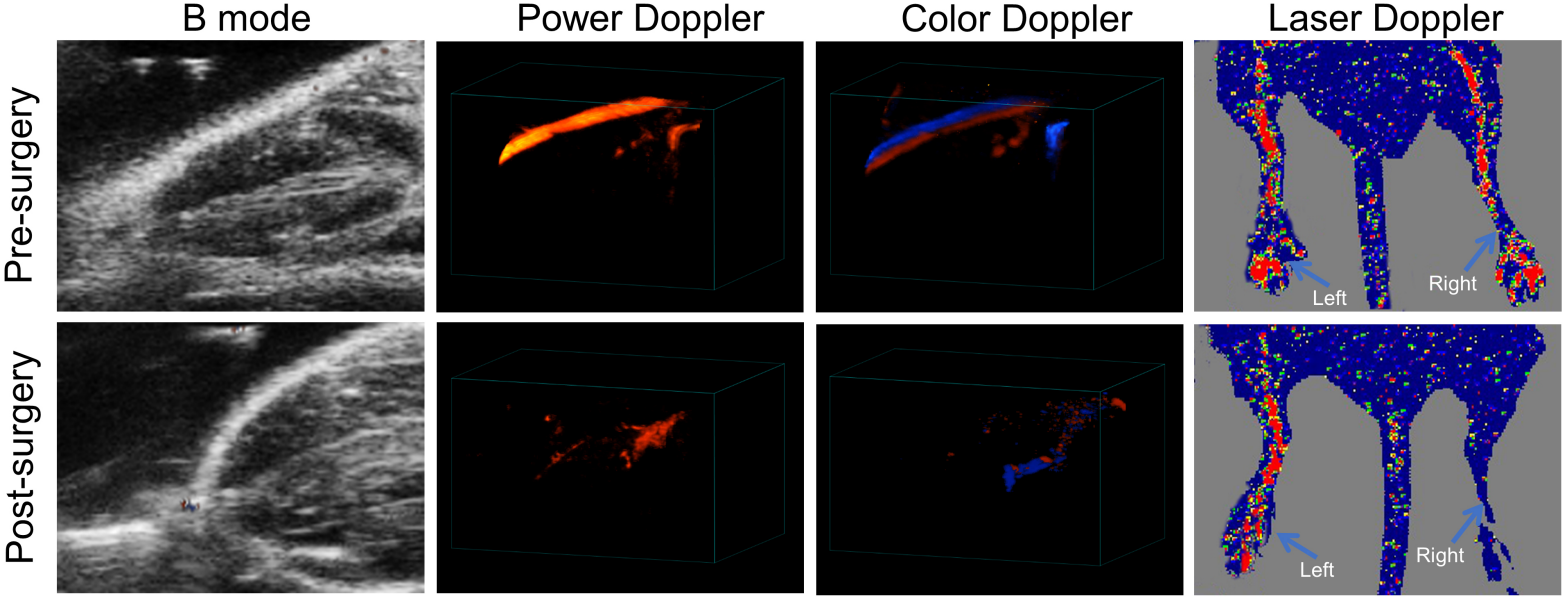
**Characterization of a murine hindlimb ischemia (HI) model **. Before surgery and one day post-surgery, blood flow in right hindlimb was detected by Power Doppler (PDI), Color Doppler (CDI) and Laser Doppler imaging (LDI), respectively. Red and blue colors acquired by CDI represents blood flow with different directions, which flows toward and away from the transducer.

Firstly, we established a murine HI model to mimic PAD. Unilateral HI was induced by ligation and excision of the right femoral artery as well as excision of its side branches. Subsequently, blood perfusion in the hindlimb was detected by multiple imaging modalities, including Laser Doppler perfusion imaging (LDPI), Power Doppler imaging (PDI), and Color Doppler imaging (CDI), all of which showed the blood flow within the right hindlimb was precipitously reduced post surgery, as compared to blood flow before surgery (Fig. 1). Additionally, both PDI and CDI revealed the lack of side branches originating from the stem femoral artery after surgery which were intact before surgery (Fig. 1). These results confirmed the successful establishment of the murine HI model.

### 3.2 Binding Specificity of ^18^F-PRGD2 to Integrin Receptor

**Fig. 2.**
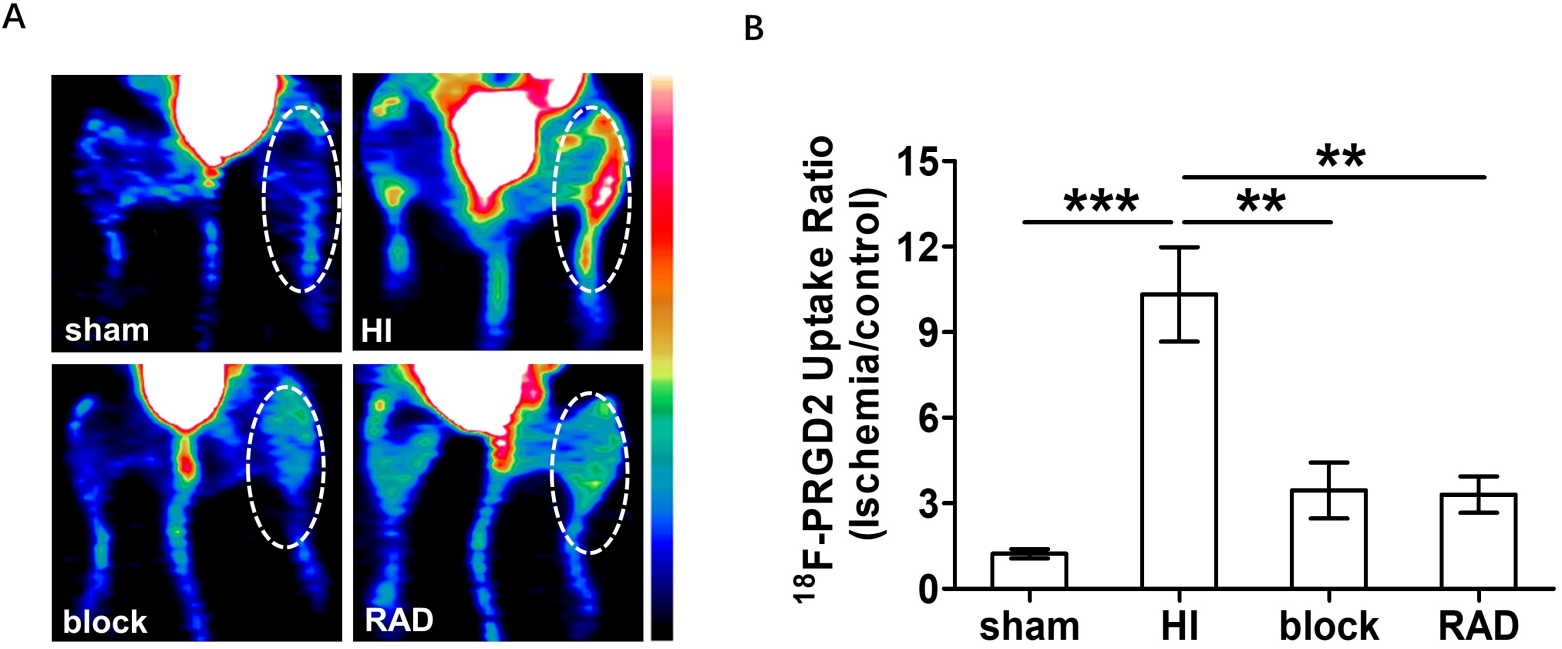
**Specificity of integrin-targeted PET imaging**. (A) Representative transaxial PET images using ^18^F-AlF-NOTA-PRGD2 (^18^F-PRGD2) (HI: 10.33 ± 3.707, n = 5), or with a block reagent (block: 3.45 ± 2.168, n = 5), or ^18^F-AlF-NOTA-RAD (RAD: 3.308 ± 1.431, n = 5) at 7 days after HI surgery. ^18^F-PRGD2 was also used in mice which underwent a sham operation (sham: 1.227 ± 0.368, n = 5). (B) Quantification of ^18^F-PRGD2 uptake ratio (ischemic/control hindlimb) by PET at different groups. **p < 0.01; ***p < 0.001.

The chemical structure of ^18^F-AlF-NOTA-PRGD2 (^18^F-PRGD2) is shown in **Supplementary Fig. 1**. When the reaction volume was maintained between 50 and 100 μL, the labeling yield was about 20–25%. Without HPLC purification, the total synthesis time was reduced to 25 minutes. The radiochemical purity was over 97%. Fig. 2 showed significant increase of focal tracer retention at 7 days after onset of ischemia (10.33 ± 3.567) compared to sham (1.227 ± 0.368). In order to further confirm the binding specificity of ^18^F-PRGD2 to integrin receptor, we utilized non-radiolabeled integrin-specific ligand, ${\mathrm{E[c(RGDyK)]}}_{2}$, as a blocking agent. *In vivo* PET imaging demonstrated that the ^18^F-PRGD2 uptake ratio in the blocked group (3.45 ± 2.186) was markedly lower than that in the unblocked group one week post HI, indicating the tracer accumulation in the ischemic skeletal muscle could be prevented by excess amount of unlabeled RGD peptide. A similar procedure was also performed to label a RAD peptide. After using this labeled RAD peptide, we found that the tracer uptake (3.308 ± 1.431) was significantly lower than that of ^18^F-PRGD2 at 7 days after HI (Fig. 2B). The low uptake of the control peptide supports specific binding of ^18^F-PRGD2 to the ischemic area rather than nonspecific leakage through injured vasculature. These results prove the specificity of integrin-targeted imaging.

### 3.3 In Vivo PET Imaging of Angiogenesis Induced by HI

**Fig. 3.**
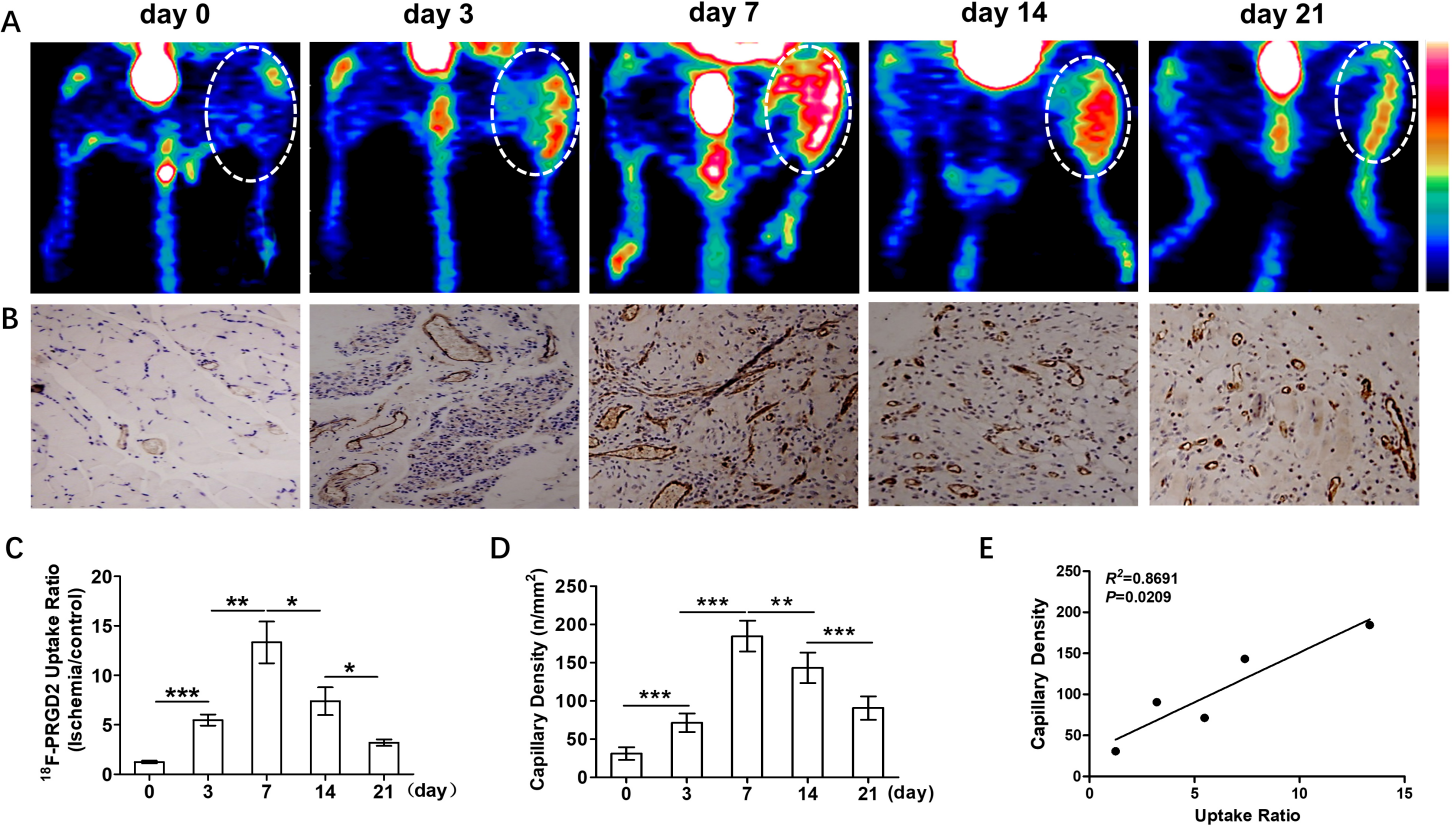
**Close correlation between angiogenesis and ^18^F-PRGD2 uptake**. (A) Representative transaxial PET images using (^18^F-PRGD2) at day 0 (pre-surgery) and days 3, 7, 14, and 21 post surgery, respectively. (B) CD31 positive vessels were immunohistochemically detected on day 0 and days 3, 7, 14 and 21 post-surgery, respectively. (C) Analysis of ^18^F-PRGD2 uptake ratio (ischemic/control hindlimb) by PET at a serial of time points (day 0: 1.249 ± 0.296, n = 6; day 3: 5.483 ± 1.354, n = 6; day 7: 13.34 ± 5.169, n = 6; day 14: 7.397 ± 3.434, n = 6; day 21: 3.2 ± 0.804, n = 6). (D) Quantitative analysis of CD31 capillary density at a series of time points post-surgery (day 0: 30.83 ± 8.159, n = 6; day 3: 71.33 ± 12.01, n = 6; day 7: 184.5 ± 20.21, n = 6; day 14: 143.2 ± 20.11, n = 6; day 21: 90.5 ± 15.1, n = 6). (E) Correlation analysis between ^18^F-PRGD2 uptake ratio (ischemic/control) and angiogenic activity, which is represented by capillary numbers (*$R^{2}$* = 0.8691, *p* = 0.0209). *p < 0.05; **p < 0.01; ***p < 0.001. Scale bar: 100 μm.

To monitor angiogenesis after HI, longitudinal PET imaging using ^18^F-PRGD2 probe was performed at serial time points including day 0 (pre-surgery) and at days 3, 7, 14, and 21 post surgery. Fig. 3A shows representative transaxial PET images with ^18^F-PRGD2. Compared with day 0, an increase of ^18^F-PRGD2 tracer uptake could be quickly detected as early as day 3 after HI and reached a maximum at day 7, which was consistent with most previous studies [17, 24]. The signal then gradually reduced in the following days. The quantitative results based on PET imaging are presented in Fig. 3C. The hindlimb uptake ratio of ^18^F-PRGD2 in the pre-surgery group was minimal (1.249 ± 0.296). HI injury led to a significant increase in uptake ratio of ^18^F-PRGD2 in the ischemic hindlimb at 3 days (5.483 ± 1.354) post surgery. Radiotracer localization after onset of ischemia was maximal at 7 days (13.34 ± 5.169). Although decreased by 3 weeks, the uptake of the radiotracer was still greater than that in the pre-surgery group.

Skeletal muscle tissues were also harvested at various time points before and after surgery for histological analysis. IHC staining was performed using CD31, a maker for endothelial cells. As shown in Fig. 3B, numerous neutrophils (nuclei in blue or purple) were infiltrated from the damaged lumen, evidenced by discontinuous endothelial layer (highlighted by brown color). These signs indicated intense inflammation in this period. Thereafter, angiogenesis was robustly activated and reached a peak at day 7 (184.5 ± 20.21), as quantified by CD31 staining (Fig. 3D). In the following two weeks, angiogenesis continued within ischemic muscle, yet with gradually reduced activity.

Additionally, correlation analysis was also performed between ^18^F-PRGD2 uptake ratio (ischemic/control) and angiogenesis, which is indicated by capillary numbers. As demonstrated in Fig. 3E, there was a strong correlation between these two variables (*p* = 0.0209). On the basis of the similar temporal changes and positive correlation between angiogenic activity and ^18^F-PRGD2 uptake ratio, we believe that the fluctuation of ^18^F-PRGD2 uptake was able to reflect the variation of angiogenesis in ischemic tissue.

### 3.4 In Vivo Assessment of Angiogenic Response to Therapy

**Fig. 4.**
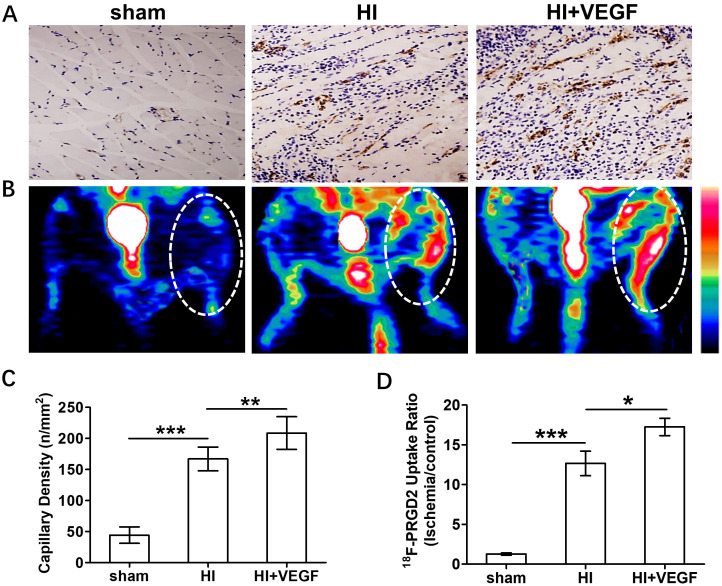
**Evaluation of VEGF treatment on angiogenic activity**. (A) *Ex vivo* immunohistochemical (IHC) staining of CD31 to examine angiogenesis in the ischemic hindlimb tissue sections 7 days post surgery in the sham-operated groups (sham), no-treatment (HI) and VEGF-treated group (HI + VEGF), respectively. (B) *In vivo* noninvasive PET imaging of angiogenesis at 7 days after surgery in different groups. (C) Quantitative analysis of CD31 capillary density (sham: 44.33 ± 13.29, n = 6; HI: 167 ± 19.24, n = 6; HI + VEGF: 208.7 ± 26.28, n = 6). (D) Quantification of ^18^F-PRGD2 uptake ratio (ischemic/control hindlimb) from *in vivo* PET imaging (sham: 1.269 ± 0.325, n = 5; HI: 12.67 ± 3.438, n = 5; HI + VEGF: 17.25 ± 2.441, n = 5). *p < 0.05; **p < 0.01; ***p < 0.001. Scale bar: 100 μm.

As one of the most established angiogenesis stimuli, VEGF was chosen in the present study for HI treatment. Compared with the non-treated HI group at 7 days post-surgery (167 ± 19.24), newly formed capillaries stained with CD31 were found to be elevated after treated with VEGF (208.7 ± 26.28) (Fig. 4A,C). Given the fact that the alteration of ^18^F-PRGD2 uptake ratio is positively correlated with angiogenic activity during HI, we then determined whether PET imaging with ^18^F-PRGD2 probe could be applied to evaluate the angiogenic response to therapy. As Fig. 4B showed, the signal intensity in the region of ischemic hindlimbs in the VEGF-treated group was significantly stronger than in the non-treated group. Based on the quantification of PET imaging, the ^18^F-PRGD2 uptake ratio of ischemic region in the VEGF-treated group (17.25 ± 2.441) was nearly fifteen times higher than that in the sham group (1.269 ± 0.325), while the uptake ratio of ischemic region in the non-treated group (12.67 ± 3.438) was only about ten times higher than that in the sham group (Fig. 4D).

**Fig. 5.**
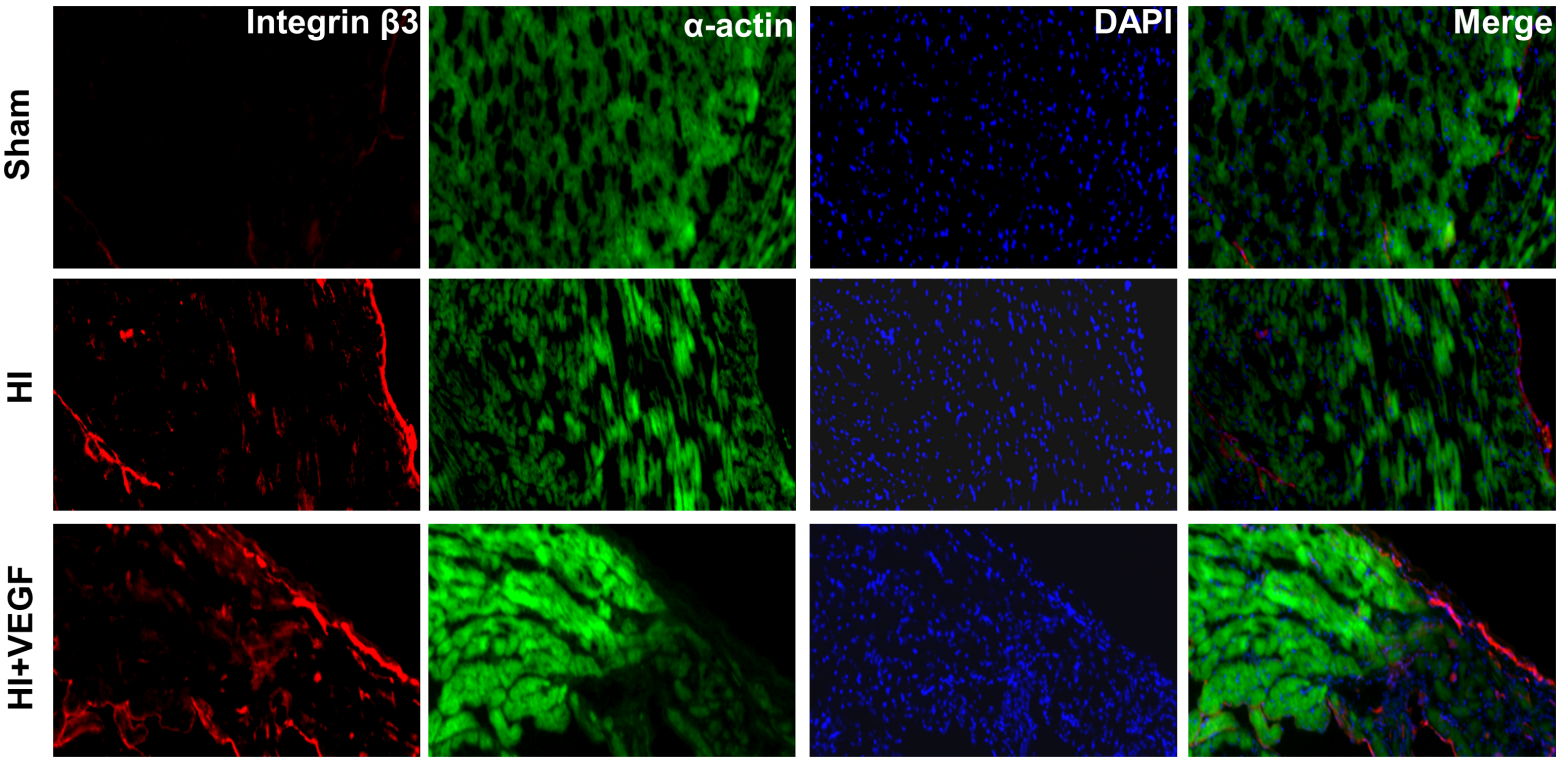
**Immunofluorescence staining of integrin β3 in the VEGF-treated (HI + VEGF), no-treatment (HI) and sham-operated groups (sham)**. Scale bar: 200 μm.

According to the immunofluorescence staining, the integrin β3 level in the VEGF-treated group (HI + VEGF) was higher than in both saline-treated group (HI) and sham group (sham) (Fig. 5), which was consistent with the pattern of radioactive signal as demonstrated by *in vivo* PET imaging. These results suggested that the ^18^F-PRGD2 uptake ratio was positively correlated with integrin β3 level within ischemic area and it could serve as a tool for noninvasive evaluation of the HI treatment response.

## 4. Discussion

In the present study, we reported for first time the application of a one-step labeled PET tracer ^18^F-AlF-NOTA-PRGD2 (^18^F-PRGD2), for the noninvasive monitoring of temporal changes of α_v_β_3_ integrin level during ischemia-induced angiogenesis in a mouse HI model. In addition, we used this tracer to evaluate the angiogenic response to VEGF treatment.

Although molecular imaging techniques have not yet reached the standard for clinical application for imaging peripheral angiogenesis, extensive preclinical work has demonstrated that noninvasive serial analysis of angiogenesis is achievable and holds great promise for clinical translation with primary targets including α_v_β_3_ integrin [25]. Lee *et al*. [26] used iodine-125 (^125^I)-labeled RGD peptides for α_v_β_3_ integrin targeting in mouse HI model with SPECT imaging and observed that radiotracer uptake reached a peak on day 3 and was maintained at a relatively lower level at 8 and 14 days of ischemia, but still higher than at the onset of ischemia. Furthermore, serial changes of α_v_β_3_ integrin expression were also noninvasively assessed with ^99^mTc-NC100692 probe during the angiogenic process in the ischemic hindlimb. Imaging results demonstrated a significantly enhanced radiotracer uptake at 3 days and a peak at 7 days [24]. In addition, Jeong *et al*. [17] used ^68^Ga-NOTA-RGD as the PET imaging tracer and investigated its biodistribution at 7 days after femoral artery ablation in mice and demonstrated its high affinity for the α_v_β_3_ integrin and specific uptake by angiogenic hindlimb tissue. In the current study, for the first time, we applied a one-step labeled PET tracer ^18^F-PRGD2 to evaluate the temporal changes of α_v_β_3_ integrin expression in mouse hindlimb tissue exposed to ischemia. Our imaging results with ^18^F-PRGD2 displayed a similar pattern of tracer uptake in the ischemic region as in earlier work. The significant increase of focal tracer retention could be quickly observed at 3 days post ischemia. Apart from the formation of newly regenerated capillaries at this time, shown by CD31 staining, intensive inflammation occurred after HI which also accounted for the high uptake of ^18^F-PRGD2 since α_v_β_3_ integrin is highly expressed on infiltrating macrophages [27]. Indeed, IHC staining (Fig. 3B) revealed that a large number of inflammatory cells had infiltrated among affected skeletal muscle on day 3 after surgery. The tracer accumulation reached a maximum on day 7. Thereafter, ^18^F-PRGD2 uptake within the ischemic hindlimb decreased from the peak level but was still higher than that in the sham-operated hindlimb, suggesting local continuous angiogenesis.

When VEGF treatment was applied, the ^18^F-PRGD2 uptake in the ischemic area was elevated and associated with increase of α_v_β_3_ integrin expression, which showed a similar tendency with angiogenesis development after HI. Our data indicated that ^18^F-PRGD2 uptake is positively correlated with angiogenic activity during HI and is able to increase in response to VEGF treatment. It could serve as a prospective probe used to monitor the angiogenic response post HI, which might help evaluate therapeutic effect of pro-angiogenesis medications treating ischemic diseases in clinical practice.

Compared to other radiolabeled tracers in previous studies, the PET tracer ^18^F-PRGD2 used here has several noticeable advantages. Firstly, when compared to traditional SPECT imaging, continued growth in the use of PET imaging in both pre-clinical and clinical work can be ascribed to increased availability of hybrid with computed tomography (CT) or magnetic resonance imaging (MRI), providing high sensitivity as well as good spatial resolution. Thus, PET radiotracers targeting angiogenesis have been investigated more extensively than SPECT radiotracers. In addition, ^18^F has been considered as an ideal radioisotope for PET imaging due to its physical properties, including short physical half-life, high positron efficiency and low β+ energy. Secondly, as a dimeric RGD peptide tracer, ^18^F-PRGD2 has better binding affinity to integrin α_v_β_3_ than RGD monomers, such as Galacto-RGD, resulting in higher integrin targeted accumulation and more favorable *in vivo* kinetics. Thirdly, compared with another previously used dimeric RGD peptide tracer ^18^F-FPPRGD2 [19], synthesis of ^18^F-PRGD2 is efficient and consequently results in relatively high labeling yield. This convenient one step route for labeling can be achieved by the displacement of the ^18^F with a leaving group in a pre-attached chelator on RGD peptides without the need of HPLC purification [18, 20]. Hence, it is reasonable to surmise that the ideal physical properties and good binding affinity as well as the simple labeling procedure would make ^18^F-PRGD2 a very promising radiotracer for clinical translation in cardiovascular diseases.

## 5. Conclusions

PET imaging of a one-step labeled integrin-targeted probe, ^18^F-AlF-NOTA-PRGD2 (^18^F-PRGD2), enables longitudinal monitoring of angiogenesis development and noninvasive assessment of VEGF treatment response in mouse model of hindlimb ischemia. The simple synthesis procedure, specific binding affinity and favorable *in vivo* performance of this PET tracer may assist in screening pro-angiogenic drugs in the preclinical setting and future clinical evaluation of ischemic lesion and therapy responses in patients with ischemic cardiovascular diseases, especially peripheral arterial disease.

## Supplementary Material

Supplementary material associated with this article can be found, in the online version, at https://doi.org/10.31083/j.rcm2312408.
