# Supplementary figures and images for: Noninvasive Evaluation of Angiogenesis and Therapeutic Response after Hindlimb Ischemia with an Integrin-Targeted Tracer by PET
Source: Rev Cardiovasc Med. 2022 Dec 14;23(12):408. doi: 10.31083/j.rcm2312408 (PMC11270400; doi:10.31083/j.rcm2312408)

Supplementary Figure


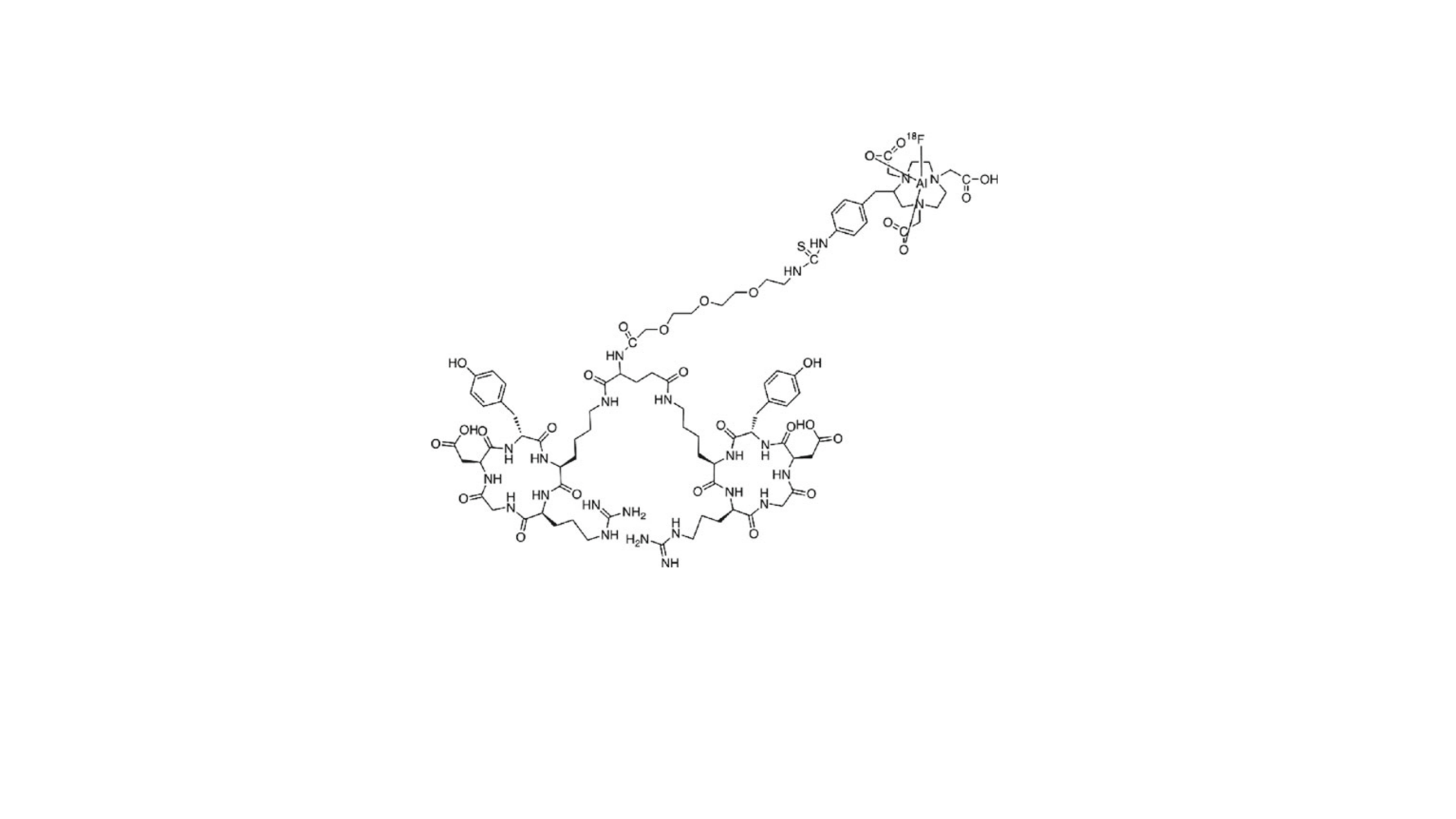


Supplementary Fig. 1. Chemical structure of 18F-AlF-NOTA-PRGD2 (18F- PRGD2).

Supplement: Supplementary file 1 [file 2153-8174-23-12-408-s1.docx]
